# Haemophilin-Producing Strains of *Haemophilus haemolyticus* Protect Respiratory Epithelia from NTHi Colonisation and Internalisation

**DOI:** 10.3390/pathogens10010029

**Published:** 2021-01-01

**Authors:** Brianna Atto, Dale Kunde, David A Gell, Stephen Tristram

**Affiliations:** 1 School of Health Sciences, University of Tasmania, Newnham Drive, Launceston, TAS 7248, Australia; dale.kunde@utas.edu.au; 2 School of Medicine, University of Tasmania, 17 Liverpool Street, Hobart, TAS 7000, Australia; david.gell@utas.edu.au

**Keywords:** *Haemophilus haemolyticus*, *Haemophilus influenzae*, haem, haem-binding protein, haemophore, haemophilin, host–cell interactions, respiratory probiotic, respiratory infections

## Abstract

Nontypeable *Haemophilus influenzae* (NTHi) is a significant respiratory tract pathogen responsible for infections that collectively pose a substantial health and socioeconomic burden. The clinical course of these infections is largely dictated by NTHi interactions with host respiratory epithelia, and thus, approaches that disrupt colonisation and invasion may have significant therapeutic potential. Survival, successful host–cell interactions, and pathogenesis are reliant on NTHi’s ability to sequester host-derived haem. Previously, we demonstrated the therapeutic potential of exploiting this haem-dependence using a closely related competitor bacterium, *Haemophilus haemolyticus* (Hh). Hh strains capable of producing the novel haem-binding protein haemophilin (Hpl) possessed potent inhibitory activity by restricting NTHi access to haem in a broth co-culture environment. Here, we extend this work to cell culture models that more closely represent the human respiratory epithelium and show that Hh strains with high levels of *hpl* expression protect epithelial cell line monolayers against adhesion and invasion by NTHi. Inhibitory activity was dependent on the level of Hpl production, which was stimulated by NTHi challenge and nasopharyngeal cell exposure. Provided these protective benefits translate to in vivo applications, Hpl-producing Hh may have probiotic utility against NTHi infections by inhibiting requisite nasopharyngeal colonisation.

## 1. Introduction

Nontypeable *Haemophilus influenzae* (NTHi) is commonly associated with nasopharyngeal colonisation in healthy children and adults but is considered an important opportunistic pathogen in predisposed individuals [1]. Common infections include otitis media (OM) in children, pneumonia in the elderly, and exacerbations of underlying lung diseases, such as chronic obstructive pulmonary disease (COPD) [2]. Despite the significant health and socioeconomic burdens associated with NTHi infections, there are currently no effective vaccination strategies available, and the treatment of existing infections is compromised by the rapid development of resistance to first- and second-line antibiotics [2,3,4,5,6,7]. 

The course of NTHi infection is largely dictated by bacterial interactions with host respiratory epithelial cells. Attachment to respiratory epithelial cells in the nasopharynx is a pre-requisite for colonisation and subsequent bacterial migration to, and infection at other sites in the respiratory tract [8]. Although the mechanisms that lead from colonisation to infection are poorly understood, a high density of NTHi carriage is associated with an increased risk of development of OM [9,10,11] and a clinically significant increase in respiratory symptoms in COPD, even in the absence of a clinical exacerbation [12]. Following successful attachment, NTHi has demonstrated the ability to invade nasopharyngeal, alveolar, bronchial, and laryngeal cell lines in vitro [8,13,14,15,16,17]. Intraepithelial NTHi are protected from bactericidal antibodies and high antibiotic concentrations [8,18], which are characteristics shown to potentiate infection severity and treatment failure, resulting in bacterial persistence and recurrent infections in COPD airways and the middle ear [13,19,20,21,22]. As such, intracellular NTHi are considered a major determinant of pulmonary morbidity in COPD patients [23] and may contribute to the prolonged and intractable clinical course of acute OM [13]. 

Successful colonisation and survival within the host relies heavily on NTHi’s ability to acquire and utilise iron-containing haem [24,25,26,27]. Although NTHi lacks the necessary enzymes required for de novo haem synthesis, it possesses a ferrochelatase that inserts iron into protoporphyrin IX to form haem [28]. Thus, NTHi can fulfil haem requirements by acquiring iron in the presence of protoporphyrin or by scavenging haem bound to host haemoproteins. For this reason, NTHi expresses a complex and redundant set of haem acquisition pathways to overcome host nutritional immunity in an environment considered to have an inherently low abundance of haem [29]. 

As well as being an absolute growth requirement, there is also evidence that haem-scavenging machinery plays a role in pathogenesis by the modulation of virulence factors, antimicrobial resistance, immune evasion, and host–cell interplay [30,31,32,33]. Haem-acquisition/utilisation genes have a higher prevalence in NTHi isolated from OM infection, compared to colonising throat strains [27] and are upregulated during infection of the chinchilla middle ear [34]. Deletion of these genes reduces the bacterium’s capacity to colonise and persist within the nasopharynx, resulting in an infection with attenuated disease severity and duration in animal models of OM and airway infection [31,33,35,36]. Similarly, an isogenic mutant of two haem-acquisition pathways was unable to sustain bacteraemia or produce meningitis in a rat model of invasive disease [35]. Thus, restricting haem acquisition or utilisation is a potentially high-value target for the development of novel therapies for the prevention of colonisation with, or eradication of NTHi from the respiratory tract [21,37]. However, due to the highly redundant nature of these pathways, approaches that target multiple haem sources or acquisition pathways are likely required to induce sufficient nutritional starvation to elicit a therapeutic response [32]. 

Previously, we have shown that a closely related bacterium, *Haemophilus haemolyticus* (Hh), competes with NTHi for limited haem resources. Hh strains capable of producing the novel haem-binding protein haemophilin (Hpl) possessed potent inhibitory activity by restricting NTHi access to haem in vitro [38,39,40]. These findings suggest that Hh strains capable of producing high levels of Hpl might disrupt NTHi association with airway epithelial cells and have potential as a probiotic that prevents the requisite nasopharyngeal colonisation stage of infection. Here, we explore this question using Hh strains with different levels of *hpl* expression in cell culture models of nasopharyngeal and lung epithelial cells.

## 2. Results

### 2.1. Cell Monolayers Pre-Treated with Hpl-Producing Strains of Hh Were Protected from NTHi Attachment and Invasion 

To investigate interactions of Hh and NTHi with epithelial cells, we used two cultured cell lines, D562 human pharyngeal epithelial cells and A549 human lung epithelial cells. Initial experiments were performed to determine the time course for the adhesion of each Hh or NTHi isolate to epithelial cells. Maximum attachment for all Hh strains was achieved after 4-h incubation (Figure S1). In comparison, NTHi strains attached in greater numbers and required only 1 h or 2 h to reach maximum attachment to A549 and D562 cells, respectively (Figure S1). Similar inter- and intra-species variation in adhesion to respiratory cells has been reported previously [13,14,15,16]. Hh and NTHi viability in both cell culture media was maintained for up to 8 h (Figure S3), and A549 and D562 cell lines did not show any substantial changes in viability for up to 8-h post challenge with Hh or NTHi strains compared to media alone (Figure S4).

**Figure 1:**
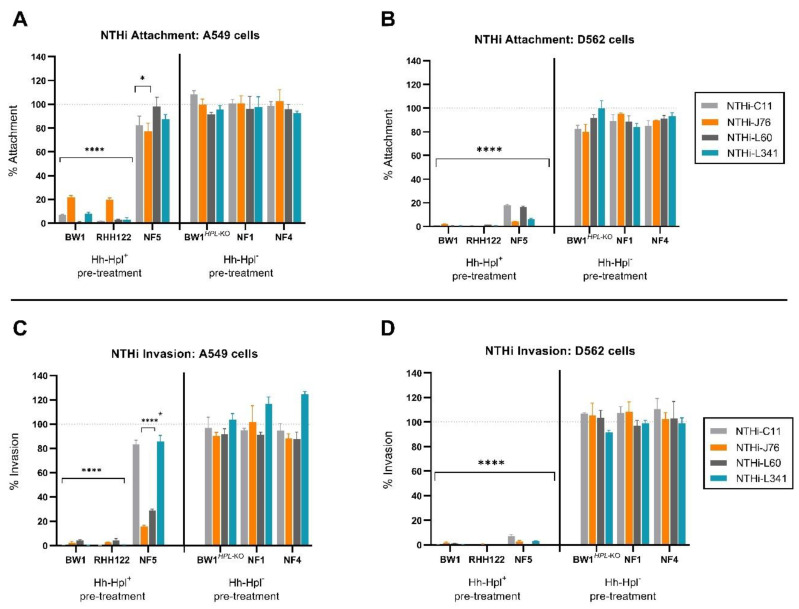
Nontypeable *Haemophilus influenzae* (NTHi) attachment and invasion of A549 and D652 cells post *Haemophilus haemolyticus* (Hh) treatment. The percent attachment of NTHi (compared to media control) to A549 (**A**) and D562 (**B**) cell monolayers post 4-h pre-treatment with Hpl-producing Hh (Hh-Hpl^+^) or Hh strains that do not produce Hpl (Hh-Hpl^−^). Percent of internalised NTHi (compared to media control) after exposure to A549 (**C**) and D562 (**D**) cell monolayers post 4-h pre-treatment with Hh-Hpl^+^ or Hh-Hpl^−^. Error bars represent the ±SEM of three biological replicates, measured triplicate: * *p*<0.05, **** *p* < 0.0001.

Based on these experiments, epithelial cell monolayers were incubated with Hh strains for 4 h prior to challenge with NTHi strains, and the number of Hh cells in the inoculum was set (Table S1) to achieve approximately equal attachment of each Hh strain at the 4-h time point (Figure S5). Following the pre-incubation with Hh, the cell cultures were challenged with a standard dose of NTHi for 1 h (A549 cells) or 2 h (D562 cells) and the number of Hh and NTHi attached to the surface or internalised within the epithelial cells was determined by colony enumeration on appropriate selective media. Cell monolayers pre-treated with Hh-BW1 or Hh-RHH122 experienced a significant 78.1–99.1% and 98.3–99.5% decrease in attachment of all four NTHi strains tested in the case of A549 (Figure 1A, *p* < 0.0001) and D562 (Figure 1B, *p* < 0.0001) cells, respectively. Pre-treatment with Hh-NF5 significantly inhibited the attachment of all NTHi strains to D562 cells (82.0–95.6%, *p* < 0.001) but offered limited or no protection in the case of A549 cell monolayers. Pre-treatment with Hh-NF4, Hh-NF1, or Hh-BW1*^Hpl^*^-KO^ did not significantly affect NTHi attachment to either cell line (*p* > 0.05). 

In the absence of Hh pre-incubation, strains NTHi-C11 and NTHi-L341 demonstrated strong invasive capacity, whereas other NTHi and Hh strains used in this study were considered non-invasive (Figure S2), which is defined as the colony-forming units (CFU) of internalised bacteria accounting for less than 1% of the original inoculum [15,41]. After 4 h pre-incubation with Hh-BW1 or Hh-RHH122, the invasive capacity of all NTHi, including highly invasive NTHi-C11 and NTHi-L341 strains, was significantly reduced (Figure 1C,D) to levels around that of the non-invasive strains (data not shown). Pre-treatment with Hh-NF5 inhibited NTHi-C11 and NTHi-L341 invasion of one cell line (D562), whereas pre-treatment with Hh-NF4, Hh-NF1, or Hh-BW1*^Hpl^*^-KO^ did not affect NTHi invasion. These experiments demonstrate considerable variation in the ability of different Hh isolates to inhibit the invasion of model epithelium cell lines by NTHi.

### 2.2. Small Treatment Doses of Hh-BW1 or Hh-RHH122 Were Sufficient to Inhibit NTHi Interactions with Model Epithelium Cell Lines

**Figure 2:**
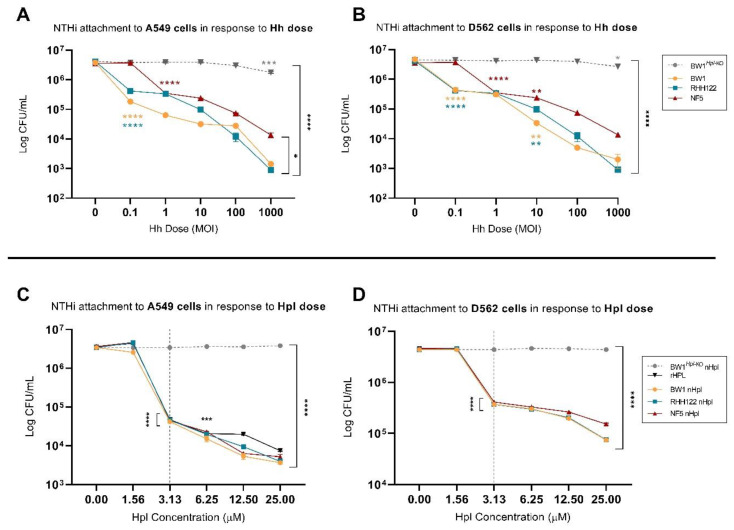
NTHi attachment to A549 and D652 cells treated with varying doses of haemophilin (Hpl)-producing Hh or purified Hpl. Colony counts (colony-forming units (CFU)/mL) of NTHi attached to A549 (A) and D562 (B) cell monolayers post 4-h pre-treatment with varying doses of Hpl-producing Hh or 2-fold increasing concentrations of native (nHpl) or recombinant Hpl (rHpl) (C,D). Error bars represent the ±SEM of three biological replicates. * *p* < 0.05, ** *p* < 0.005 *** *p* < 0.001, **** *p* < 0.0001.

**Figure 3:**
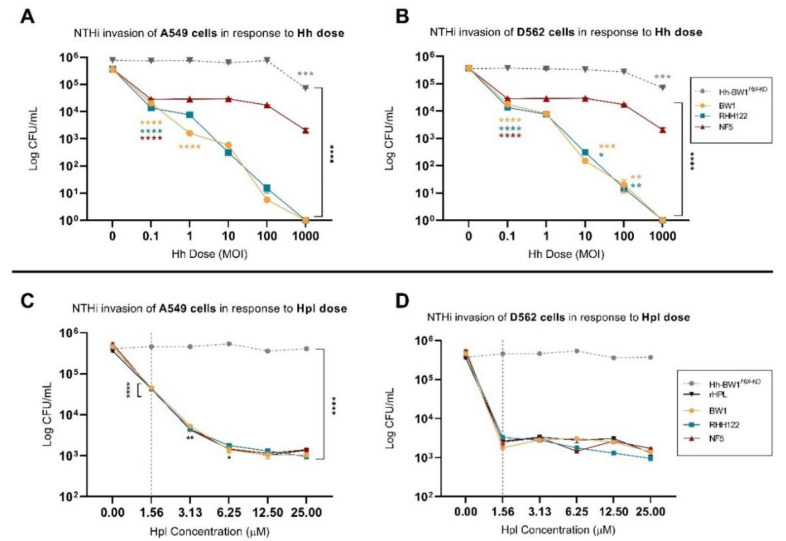
**NTHi internalisation in A549 and D652 cells treated with different doses of Hpl-producing Hh or Hpl.** Colony counts (CFU/mL) of internalised NTHi in A549 (**A**) and D562 (**B**) cell monolayers post 4-h pre-treatment with varying doses of Hpl-producing Hh or 2-fold increasing concentrations of native (nHpl) or recombinant Hpl (rHpl) (**C**,**D**). Error bars represent the ±SEM of three biological replicates. * *p* < 0.05, ** *p* < 0.005 *** *p* < 0.001, **** *p* < 0.0001.

To investigate the relative protective potency of Hh strains with different levels of *hpl* expression, cell monolayers were pre-treated with varying doses of Hh-BW1, Hh-RHH122, Hh-NF5, or Hh-BW1*^Hpl-KO^* prior to NTHi challenge. NTHi-C11 was selected as the competitor strain based on its overall superior attachment (Figure S1**)** and invasive capacity (Figure S2). Pre-treatment with Hh-BW1 or Hh-RHH122 at an Hh:NTHi ratio of 0.1:1 resulted in a 90.0–95.2% reduction in NTHi attachment to A549 cells (Figure 2A, *p* < 0.0001) and a reduction of 90.0–90.6% to D562 cells (Figure 2B, *p* < 0.0001). At the highest treatment dose (Hh:NTHi ratio 1000:1), pre-treatment with Hh-BW1 and Hh-RHH122 resulted in a 99.0 ± 0.2% inhibition of NTHi attachment compared to pre-treatment with media alone. Pre-treatment with Hh-NF5 required approximately 10-fold higher treatment load to achieve the same degree of NTHi inhibition as Hh-BW1 or Hh-RHH122 at the low and high Hh:NTHi treatment ratios. A similar pattern of inhibition was seen for NTHi invasion (Figure 3A, B). NTHi invasion of cell monolayers was decreased by 92.3–96.3% following pre-incubation with the lowest doses of Hh-BW1 or Hh-RHH122, and no internalised NTHi were found at the highest Hh:NTHi ratio (1000:1). Preincubation with Hh-NF5 gave lower protection against NTHi invasion than Hh-BW1 or Hh-RHH122 at all except the lowest challenge ratio. Except for the highest treatment load (Hh:NTHi ratio 1000:1), no significant reduction in attachment or invasion of NTHi was observed in cell monolayers treated with Hh-BW1*^Hpl^*^-KO^. 

### 2.3. Purified Hpl Inhibits NTHi Interactions with Model Epithelium Cell Lines

In the above experiments, Hh strains previously shown to be high-level *hpl* expressors were more effective at inhibiting NTHi attachment and invasion than other Hh, and the loss of this protective effect in Hh-BW1*^Hpl^*^-KO^ strongly implicates a mechanistic role for Hpl. However, other factors, such as differential expression of cell surface adhesins and physical interactions between bacterial cells could also be important. To investigate a direct role for secreted Hpl, we challenged cell monolayers with NTHi after pre-incubation with recombinant Hpl (rHpl) or cell-free supernatants from Hh cultures. Cell-free supernatants were concentrated by ammonium sulfate precipitation and the concentration of native Hpl (nHpl) was determined by bioassay using purified rHpl as the standard (Figure S6). NTHi attachment to cell monolayers was significantly reduced following treatment with purified rHpl and supernatants (nHpl) from Hpl-producing strains (Hh-BW1, Hh-RHH122, and Hh-NF5) but not control extracts from Hh-BW1*^Hpl^*^-KO^ (Figure 2C,D). The minimum concentration of Hpl required for a significant reduction in attachment was 3.1 µM (*p* < 0.0001). The NTHi invasion assay was more sensitive to Hpl, only requiring a concentration of 1.6 µM for significant inhibition (Figure 3C,D). Higher doses of Hpl reduced NTHi invasive capacity to a level similar to that of the non-invasive strains (data not shown) but, unlike treatment with Hpl-producing Hh bacteria, Hpl protein alone did not completely eradicate NTHi invasion. These experiments suggest that Hpl protein is sufficient to cause a substantial reduction in the adherence and invasion capacity of NTHi, but that additional inhibitory mechanisms occur in the presence of Hh cells.

### 2.4. Expression of Hpl Is Stimulated in Response to D562 Cell Culture and NTHi Challenge

**Figure 4:**
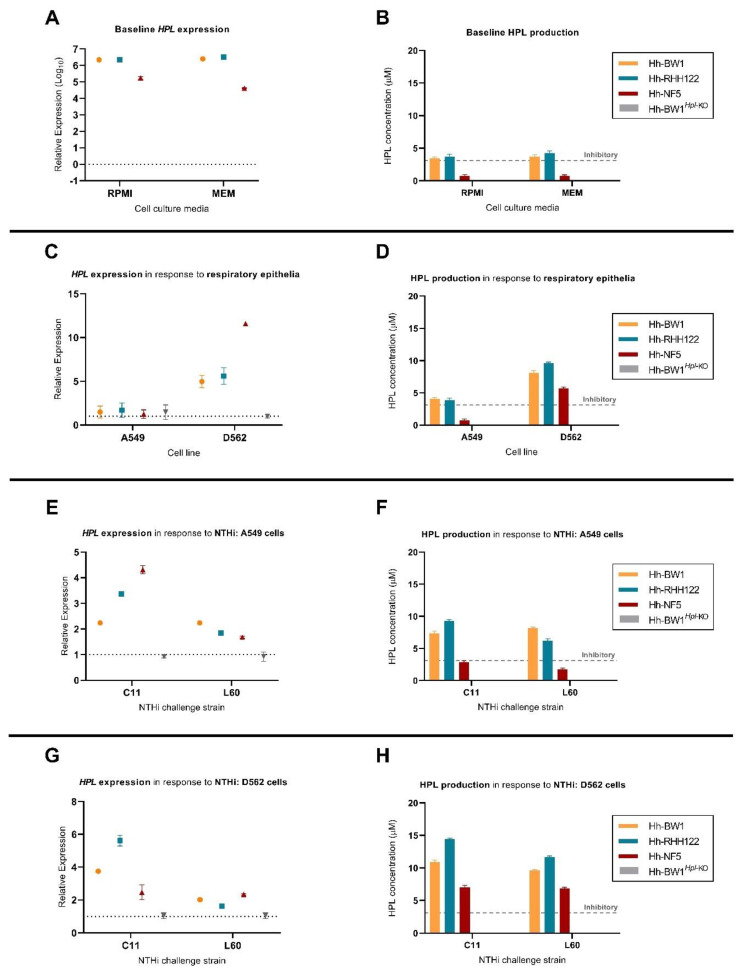
**Parallel *hpl* mRNA expression analysis and semi-quantification of Hpl protein production.** Baseline expression of hpl mRNA and protein production, recovered from 1-h incubation in cell culture media (**A**,**B**) or attached to cell monolayers relative to Hh-BW1*^Hpl^*^-KO^ (**C**,**D**). Expression of *hpl* and nHpl concentration measured from Hh attached to A549 (**E**,**F**) or D562 (**G**,**H**) cell monolayers following NTHi challenge. The minimum concentration of Hpl required to elicit NTHi-inhibitory activity during competition studies is marked on the y-axis by the grey “inhibitory” line. Data points are represented as mean ± SEM of three biological replicates, performed from duplicate mRNA extractions.

To investigate whether *hpl* expression is altered following exposure of Hh strains to mammalian cultured cells or NTHi, we performed an analysis of *hpl* mRNA and Hpl protein levels following co-culture experiments. Baseline *hpl* expression in cell culture media was highest in Hh-BW1 and Hh-RHH122, approximately 10-fold lower in Hh-NF5 (Figure 4A), and absent in Hh-NF4 and Hh-NF1 (data not shown), which is consistent with findings in bacterial growth medium [40]. Hpl mRNA and protein levels did not differ significantly between RPMI and MEM cell culture media (Figure 4A,B, *p* < 0.05). Final concentrations of Hpl produced in growth media by Hh-BW1 and Hh-RHH122 at baseline exceeded the minimum concentration required for NTHi growth inhibition, as determined by the agarose well diffusion assay (Figure 4B). By the same metric, Hh-NF5 supernatants contained sub-inhibitory concentrations of Hpl. Inhibitory activity was not detected from Hh-NF1, Hh-NF4 (data not shown), or Hh-BW1*^Hpl^*^-KO^ supernatants. Following exposure to D562, but not A549 cell monolayers, *hpl* expression was stimulated in Hh-BW1, Hh-RHH122, and Hh-NF5, compared to expression in media alone (*p* < 0.05, Figure 4C). Hh-NF5, which had the lowest baseline level of *hpl* expression, experienced the highest increase in *hpl* mRNA levels upon exposure to D562 cell monolayers (11.59-fold), which is more than double that of the other strains (*p* < 0.001), and this was reflected in the largest increase in Hpl protein concentration to levels that were expected to inhibit NTHi growth (Figure 4D). In addition, *hpl* expression (at the RNA and protein levels) was further upregulated after NTHi challenge, compared to cell monolayers alone (Figure 4E–H). The degree of stimulation varied significantly between strains but was generally higher in response to the highly invasive NTHi-C11 than in NTHi-L60 (*p* < 0.0001).

## 3. Discussion

We previously demonstrated the NTHi-inhibitory capacity of Hpl-producing Hh strains in a broth co-culture environment and proposed the probiotic utility of these strains against NTHi colonisation of the upper respiratory tract [40]. In the current study, we have extended this work to cell line culture models that more closely represent the human respiratory epithelium and show that some Hh strains with high levels of *hpl* expression protect epithelial cell line monolayers against adhesion and invasion by NTHi. 

Consistent with literature reports, Hh and NTHi host–cell interactions were highly time-dependant and demonstrated a high degree of inter- and intra-species variability [13,14,15,16,17]. In general, NTHi demonstrated a greater capacity to attach to and invade respiratory epithelial cell lines, compared to Hh. The superior attachment of NTHi has previously been reported [14] and may contribute to the bacterium’s success as a competitor in the nasopharyngeal niche and as a pathogen [42,43]. Cell interactions were also significantly influenced by the cell line. All NTHi and Hh strains displayed an improved ability to attach and, in the case of invasive NTHi, invade D562 cells, compared to A549 cell monolayers. Differences in susceptibility to NTHi invasion have previously been observed in bronchial epithelial and lung cell lines in vitro [15], which may suggest an adaptive preference for nasopharyngeal cells. However, influence of the different media composition or characteristics of the carcinoma-derived cell lines used in this study cannot be ruled out. Hh has previously demonstrated the ability to invade and cause cytotoxicity in respiratory epithelial cells in vitro, which are features that are generally associated with pathogenesis and persistence [8,14]. However, none of the Hh strains tested in the current study demonstrated invasive capacity to either D562 or A549 cell lines. Long-term exposure to Hh (24 h) did result in a significant loss of viability in A549 cells, which is an observation that has been made previously but remains unexplained [14]. The clinical significance or likely translation to the in vivo environment of the demonstrated in vitro cytotoxicity is unclear, particularly given the commensal nature of Hh. The lack of cytotoxicity observed in cell lines following treatment with purified Hpl suggests that Hpl is not a cause of cytotoxicity. 

In this study, we used five isolates of Hh with differing abilities to inhibit the growth of NTHi in broth co-cultures [40]. In these strains, the level of *hpl* expression correlates with the ability of the strain to inhibit adhesion and invasion of model epithelial cell lines by NTHi. Thus, Hh-BW1 and Hh-RHH122 strains with high-level *hpl* expression provided significant protection from NTHi attachment and reduced NTHi invasion to levels observed in non-invasive NTHi strains. Conversely, Hh-NF5, with relatively low *hpl* expression, only offered protection to D562 cells when pre-treated with larger inoculation doses, and strains that lacked detectable *hpl* expression (Hh-NF1, Hh-NF4) offered no protection to either cell line. In addition, the absence of protection in cell monolayers treated with the insertional deletion mutant, Hh-BW1*^Hpl-^*^KO^, suggests that Hpl plays a causative role in mediating NTHi-inhibitory activity. Purified recombinant Hpl also protected cell monolayers from NTHi adhesion and invasion. Whilst the maximum concentration of purified Hpl used (25 µM) was similar to the maximum level produced by Hh-BW1 and RH122 in the NTHi cell challenge conditions (≤15 µM), the level of protection conferred by Hh strains was always higher than with Hpl protein alone. One implication is that additional factors, such as the differential expression of surface adhesins and steric interactions between bacterial cells may also contribute to strain-specific differences in NTHi-inhibitory activity. A role, if any, for Hpl in regulating gene expression pathways involved in microbial competition or mammalian cell adhesion is a topic for future investigation. It has also to be considered that prolonged production and high local concentrations of Hpl generated by adherent Hh could be more effective than the single bolus application of Hpl protein to the culture. Nevertheless, the experiments presented here identify Hpl as an important effector of the NTHi-inhibitory effect.

We have previously shown that Hpl exhibits NTHi growth-inhibitory activity owing to its capacity to bind haem in a form that is inaccessible to NTHi [39]. Although the mechanism by which Hpl acts to protect model epithelial cell lines against invasion by NTHi is not explored in this study, it seems likely that this is also a result of limited haem availability to NTHi, resulting in growth inhibition [39,40]. NTHi lacks the necessary enzymes required for de novo haem synthesis, and the deleterious effects of haem starvation on NTHi are demonstrated by NTHi strains lacking either the HxuCBA, PE, SapABCDFZ, or HbpA-DppBCDF haem-acquisition systems. Compared to the wild type, these mutants had an attenuated ability to invade A549 airway epithelia or cause airway infection in a mouse model of lung infection, the degree of which was exacerbated when haem availability was restricted [31]. The in vivo consequence of haem starvation was demonstrated by the decreased persistence in the chinchilla middle ear and nasopharynx by NTHi lacking the SapF-mediated haem-iron acquisition pathway [33]. 

The Hh-BW1 and Hh-RHH122 strains used in this study were originally identified on the basis of NTHi-inhibitory activity in cell-free supernatants obtained from Hh monocultures, suggesting the constitutive high expression of *hpl*. Here, we show that *hpl* expression is regulated by environmental conditions, including co-culture with mammalian cells and NTHi. Baseline expression levels were highest in Hh-RHH122 and Hh-BW1 and approximately 10-fold lower in Hh-NF5. These expression levels corresponded to levels of Hpl production that were close to the minimum required concentration for NTHi inhibition, or in the case of Hh-NF5, they were significantly lower. *Hpl* mRNA and protein production levels were significantly stimulated during exposure to D562 cells, particularly in the case of Hh-NF5. This response was not replicated in A549 cells. This is particularly significant for Hh-NF5, which without stimulation appears to produce Hpl concentrations that do not exceed the minimum required concentration and translates into its inability to inhibit NTHi during association with A549 cells. The physical or chemical signal that elicits the upregulation of *hpl* remains to be investigated; candidates include iron/haem concentration and surface or secreted products from mammalian cells. Such mechanisms have been described in NTHi, where the type IV pilus, a mediator of adherence, colonisation, and in vivo persistence is upregulated in environments of low haem availability and was found to be stimulated by soluble factors released by respiratory epithelial cells [44].

The presence of NTHi was also found to be a significant stimulant of Hpl production, which is a response that varied between NTHi strain and Hh phenotype. Although there is no literature investigating the competitive haem acquisition between NTHi and Hh, there have been reports of siderophore-mediated interspecies competition for iron by other respiratory-associated microorganisms. Many bacterial species have the ability to utilise heterologous siderophores, shifting the cost of production to another organism and simultaneously sequestering iron away from the siderophore producer [45]. *Pseudomonas aeruginosa* was shown to upregulate the iron-scavenging siderophore pyoverdine under conditions of low iron availability in response to competition imposed by *Burkholderia cenocepacia* [46] or *Candida albicans* [47]. 

The strong protective capacity elicited by some Hh strains, despite the superior attachment capacity of NTHi, suggests a potential probiotic activity. Although Hh has occasionally been reported as a pathogen of sterile sites in immunocompromised patients [48], there is convincing evidence that they are not opportunistic pathogens of the respiratory tract [49,50,51]. In model epithelial cell lines, the significant inhibition of NTHi adherence and invasion was achieved with treatment doses of Hh strains Hh-BW1 and Hh-RHH122 that were 10-fold lower than the NTHi challenge. Based on these in vitro data, a translation to clinical significance can be speculated. The presence of healthy carriers of NTHi indicates that a complete eradication of NTHi is not necessary to prevent infection. Furthermore, higher NTHi nasopharyngeal carriage load is correlated with a large increase in susceptibility to OM in vivo [9,52,53,54] and an increased severity of airway inflammation, exacerbations, and daily symptoms in COPD [12,55]. These in vivo observations suggest that even small reductions in NTHi carriage might have a significant impact in reducing infection. The model designed to predict the risk of OM in children based on NTHi nasopharyngeal carriage load [9] can be used to roughly contextualise the potential clinical benefit if the level of protection conferred by Hh-RHH122 and Hh-BW1 to model cell lines, which resulted in a reduction of NTHi attachment from 3.95 − 4.64 × 10^6^ CFU to 0.9 − 2.0 × 10^3^ CFU, was translated to the in vivo situation. Using the in vivo model, this would translate into an OM-associated risk from ≈50% down to around ≈10%. Inhibition of NTHi invasion may also enhance the protective capacity and therapeutic utility of *hpl*-expressing strains, as intracellular NTHi within respiratory epithelial cells is associated with persistent airway colonisation and exacerbations of COPD [20,42] and a prolonged and intractable clinical course of acute OM in children [13]. However, our study does not consider the multiple biological haem-sources and complex bacterial communities that may be present in the in vivo nasopharyngeal niche, the importance of which require further investigation.

In conclusion, results from this study show that in the model epithelial cell culture system, Hh strains with a high-level production of the hemophore, hemophilin, have a strong protective capacity against NTHi adhesion and invasion, which are promising characteristics in the context of a probiotic therapy. Further investigation is required to assess factors that may influence the therapeutic potential of a range of *hpl* expressing clinical strains in vivo, particularly the influence of other biological haem sources and polymicrobial communities.

## 4. Materials and Methods 

### 4.1. Microbial Strains

Previously, we showed that production of the Hpl haemophore in five isolates of Hh predicted the ability of these strains to inhibit the growth of NTHi in broth co-cultures [40]. Thus, Hh strains containing *hpl* alleles have previously been isolated [38,39] and screened for *hpl* expression and NTHi-inhibitory capacity during broth co-culture [40]. Hh strains Hh-BW1, Hh-RH122, Hh-NF4, and Hh-NF5 encode identical Hpl protein sequences but differ in levels of *hpl* expression. Hh strains Hh-BW1 and Hh-RH122 had the highest expression of *hpl*, as determined by levels of *hpl* mRNA and secreted NTHi-inhibitory activity, and these strains induced a complete loss of growth in multiple NTHi strains in extended co-culture experiments. The Hh-NF4 strain had no detectible expression of *hpl* mRNA or secreted NTHi-inhibitory activity. Hh-NF1 had a truncated *hpl* ORF (non-identical to Hh-BW1) and no secreted NTHi-inhibitory activity. Hh-NF5 had intermediate levels of *hpl* mRNA and secreted Hpl protein and conferred an intermediate loss of growth in co-cultured NTHi. An *hpl* knockout (BW1*^Hpl^*^– KO^) of strain Hh-BW1 was constructed using insertional inactivation, as previously described [39]. NTHi clinical isolates C11 (sputum), J76 (eye), L60 (throat), and L341 (ear) have previously been collected and categorised as either “invasive” (C11 and L341) or “non-invasive” (L60 and J76) following infection of respiratory epithelial cells [15]. 

### 4.2. Microbial Growth Conditions and Propagation of Haem-Starved Populations

NTHi and Hh isolates were propagated from liquid nitrogen frozen glycerol stock, followed by two overnight passages on chocolate agar (CA) containing 2% (*v*/*v*) Vitox^®^ (Oxoid Ltd.) at 37 °C with 5–10% CO_2_ prior to experimentation. To replicate the predicted haem-restricted environment of the respiratory tract [29] and maximise adherence and invasive capacity [31], haem-starved populations of Hh and NTHi strains were prepared prior to cell association experiments. Bacterial suspensions of ≈0.1 OD_600_ were made in Tryptone Soy Broth (TSB; Oxoid Ltd., Basingstoke, UK) supplemented with 2% (*v*/*v*) Vitox^®^ (Oxoid Ltd.) from overnight growth on CA. Broths were incubated for 12 h at 37 °C aerobically with shaking (220 RPM), centrifuged at 4000× *g* for 5 min at 37 °C and resuspended in fresh, pre-warmed TSB to an OD_600_ of 1.0 prior to use in growth experiments. Exposure to non-growth conditions was minimised by maintaining suspensions and diluents at 37 °C.

### 4.3. Generation of Nalidixic Acid-Resistant Mutants

The differentiation of Haemophilus species was achieved by inducing nalidixic acid resistance in all NTHi isolates. Lawn plates were prepared by spreading 100–200 µL of a 0.5 McF standard suspension of either NTHi-C11, NTHi-J76, NTHi-L60, or NTHi-L341 on CA containing 1 µg/mL of nalidixic acid and incubated overnight at 37 °C with 5–10% CO_2_. Resistant colonies were purified by streaking onto fresh CA containing the same concentration of nalidixic acid as the preceding lawn plate. This process was repeated with resistant mutants by successive passages on CA containing increasing concentrations of nalidixic acid up to 8 µg/mL. 

### 4.4. Epithelial Cell Culture and Maintenance

Cell cultures were maintained in T75 flasks (Thermo Fisher Scientific, Scoresby, VIC, Australia) at 37 °C, 5% CO_2_. Immortalised Detroit 562 (D562) human pharyngeal carcinoma epithelial cells (ATCC^®^ CCL-138) were cultured in Minimal Essential Media (MEM) with Earle’s salts and supplemented with 10% foetal bovine serum (Sigma-Aldrich, North Ryde BC, NSW, Australia), 1 mM sodium pyruvate, 2 mM L-glutamine, 1 × non-essential amino acids. Immortalised A549 human lung carcinoma epithelial cells (ATCC^®^ CCL-185) were cultured in RPMI 1640 (Thermo Fisher Scientific,, Scoresby, VIC, Australia) supplemented with 10% foetal bovine serum. At confluence, cell monolayers were disrupted with 1x TrypLE (Gibco) for 15 min at 37 °C, 5% CO_2_, washed in HBSS and resuspended in their respective culture media. Cells were counted with a haemocytometer, and viability was determined using trypan blue staining. Cells were seeded into 24-well plates at 1 × 10^4^/mL and grown to confluence prior to bacterial challenge.

### 4.5. Baseline Attachment and Invasion of Respiratory Epithelial Cell Lines

The time required for maximum attachment/internalisation of bacteria on/in epithelial cells was determined for individual bacterial strains and cell lines prior to performing more complex competitive cell-association assays. Haem-starved populations of Hh or NTHi strains were diluted in supplemented MEM (D562) or RPMI (A549) cell culture media to a MOI of 100:1. Cell monolayers were treated (in triplicate) with 1 mL of each Hh or NTHi strain suspended in the appropriate media and incubated for 0.5, 1, 2, 4, or 24 h at 37 °C, 5% CO_2_. Cell monolayers were washed 3 times in HBSS to remove non-adherent populations. Cells were incubated for an additional hour in cell culture media containing 200 μg/mL gentamicin for the evaluation of intracellular bacteria, or in media alone for cells being evaluated for total bacterial association. Cell monolayers were washed 3 times in HBSS, lysed with 500 µL of 2% saponin *v*/*v* in HBSS for 15 min, disassociated from the plate surface by vigorous scraping with a pipette tip, followed by vortexing for 1 min. Collected lysates were serially diluted and 100 µL spread on CA. Colony-forming units (CFU) was determined by counting colonies from plates following 16–24-h incubation. Strains were categorised as “invasive” if the number of internalised bacteria exceeded 1% of the original inoculum [15,41]. 

### 4.6. Bacterial and Cell Viability

Bacterial and epithelial cell survivability was assessed under experimental conditions to ensure that viability was maintained during competition assays. To determine bacterial viability, haem-starved populations of Hh or NTHi strains were diluted in pre-warmed, supplemented MEM or RPMI cell culture media to a density of ≈1 × 10^6^ CFU/mL. Triplicate aliquots of 1 mL were transferred to wells of a 24-well plate and incubated for 24 hrs at 37 °C, 5% CO_2_. Aliquots of 100 µL were retrieved at 0, 1, 2, 4, 8, or 24 h and spread on CA. Colony-forming units (CFU) was determined by counting colonies from plates following 16–24-h incubation.

To determine epithelial cell viability, A549 and D562 cell monolayers were treated (in triplicate) with 1 mL of each Hh or NTHi strain suspended in the appropriate media at a MOI of 1000:1, or media alone and incubated for 24 h at 37 °C, 5% CO_2_. Hh-ATCC 33390 was included as a positive toxic control, as it has previously been shown to produce cytotoxic effects on both A549 and D562 cells in vitro [14]. At 0, 4, 8, or 24 h cell monolayers were washed with fresh media, disrupted with 1x TrypLE (Gibco) for 15 min at 37 °C, 5% CO_2_, washed in HBSS, and resuspended in their respective culture media. Cells were counted with a haemocytometer, and viability was determined using trypan blue staining.

### 4.7. Preparation and Quantification of Recombinant and Native Hpl

Methods used to prepare recombinant Hpl (rHpl) by purification from *E.coli*, and native Hpl (nHpl) by ammonium sulfate precipitation of Hh culture broths, have previously been described [38,39]. A previously validated well diffusion assay was used for the semi-quantification of nHpl [40]. A series of 2-fold dilutions of rHpl solution with a known concentration of 40 µM served as a standard and was tested alongside native extracts. The resultant relationship between zone size and concentration was used to determine the concentration of native extracts. Testing was conducted on two indicator NTHi strains (ATCC 49427 and clinical isolate NTHi-L15). Culture broths from strains negative for the *hpl* ORF (Hh ATCC 33390 and BW1*^Hpl^*^– KO^) were included as negative controls.

### 4.8. Competitive Cell Association and Invasion

Considering the high variability in attachment capacity between Hh strains, the initial Hh inoculum densities were normalised to allow for equal cell attachment amongst strains (see Table S1). Additionally, time points used for attachment and invasion measurements were individualised based on earlier baseline cell–interaction dynamics and viability studies. Cell monolayers (n = 6) were treated with standardised haem-starved populations of either Hpl-producing or non-producing strains of Hh in the appropriate cell culture media for 4 h, followed by 3 washes in HBSS. Cell monolayers with attached Hh strains were challenged with NTHi-C11, NTHi-J76, NTHi-L60, NTHi-L341, or media alone for an additional 1 h (for A549 cells) or 2 h (D562 cells). A challenge dose of ≈2.5 × 10^7^ was maintained among NTHi strains and was based on challenge doses associated with a high risk of OM infection in children [9] and in the induction of OM in mouse models [52,53]. Three replicates were used to prepare lysates for the quantification of adherent or internalised bacteria. Cell monolayers were washed 3 times in HBSS to remove non-adherent populations. Cells were incubated for an additional hour in cell culture media containing 200 ug/mL gentamicin for evaluation of intracellular bacteria, or in media alone for cells being evaluated for total bacterial association. Cell monolayers were washed 3 times in HBSS, lysed with 500 µL of 2% saponin *v*/*v* in HBSS for 15 min, disassociated from the plate surface by vigorous scraping with a pipette tip, followed by vortexing for 1 min. These lysates were serially diluted and 100 µL spread on CA and CA containing 4 µg/mL nalidixic acid. CFU of NTHi was determined by counting colonies from nalidixic acid CA plates following 16–24-h incubation. CFU of Hh was determined by subtracting colony counts on CA from those counted on paired CA with nalidixic acid. If colony counts of Hh in control wells (without NTHi challenge) varied significantly between Hh strains, the run was discarded. For the remaining three replicates, post-competition suspensions were collected for nHpl quantification by ammonium precipitation and well diffusion, and lysates (prepared as described above) were collected for analysis of *hpl* expression. 

Following the same competitive cell association protocol, two additional experiments were performed to assess the effect of varying Hh pre-treatment dose and also assess the NTHi-inhibitory capacity of Hpl independent of Hh. Cell monolayers were treated with 10-fold increasing doses of Hpl-producing strains of Hh ranging from 5 × 10^4^ − 5 × 10^8^ CFU/mL (equivalent to a Hh MOI of 0.1:1–1000:1) or 2-fold increasing doses (0–25 µM) of nHpl extracted from broth culture of Hh-BW1, Hh-BW1*^Hpl-^*^KO^ (negative control), or rHpl preparations. Following treatments, cell monolayers were challenged with NTHi-C11, which previously demonstrated consistently high attachment and invasive capacity. 

### 4.9. Expression Analysis

Lysates recovered from competitive colonisation and invasion assays were immediately added to two volumes of RNAprotect Bacteria Reagent (Qiagen, Chadstone, VIC, Australia) for stabilisation of bacterial mRNA. Baseline *hpl* expression was determined from Hh suspensions incubated for 1 h in MEM or RPMI cell culture media without eukaryote cells or NTHi challenge. Extraction of mRNA and real-time PCR quantification of *hpl* expression was determined, as previously described [40]. 

### 4.10. Statistical Analysis

Statistical analysis was performed using GraphPad Prism V7.04, 2017 (GraphPad Software, San Diego, California, USA). A two-way ANOVA with Dunnett’s multiple comparisons test was used to compare baseline NTHi adherence and invasion to cells pre-treated with either Hh strains or native Hpl extracts. The minimum dose of Hh or concentration of native Hpl extracts required to significantly inhibit NTHi adherence or invasion was determined using a two-way ANOVA with Tukey’s multiple comparisons test. Expression ratios and statistical significance were calculated with 2000 iterations by the Relative Expression Software Tool (REST; v 1.0, 2009) [56,57].

## Data Availability

The data presented in this study are contained within the article or supplementary materials.

## Supplementary Materials

The following are available online at https://www.mdpi.com/2076-0817/10/1/29/s1, Figure S1: Baseline bacterial attachment to epithelial cells, Figure S2: Baseline bacterial invasion of epithelial cells, Figure S3: Bacterial viability in cell culture media, Figure S4: Viability of respiratory epithelium post bacterial challenge, Figure S5: Hh attachment and invasion of A549 and D562 cells post NTHi challenge, Figure S6: Bioactivity of recombinant and native Hpl, Table S1: Specific Hh treatment load (CFU/mL) for competitive colonization experiments, standardized for differences in attachment capacity.
