# Supplementary material for: Haemophilin-Producing Strains of Haemophilus haemolyticus Protect Respiratory Epithelia from NTHi Colonisation and Internalisation
Source: Pathogens. 2021 Jan 1;10(1):29. doi: 10.3390/pathogens10010029 (PMC7823694; doi:10.3390/pathogens10010029)
Supplement: Supplementary file 1 [file pathogens-10-00029-s001.pdf]

SUPPLEMENTARY DATA

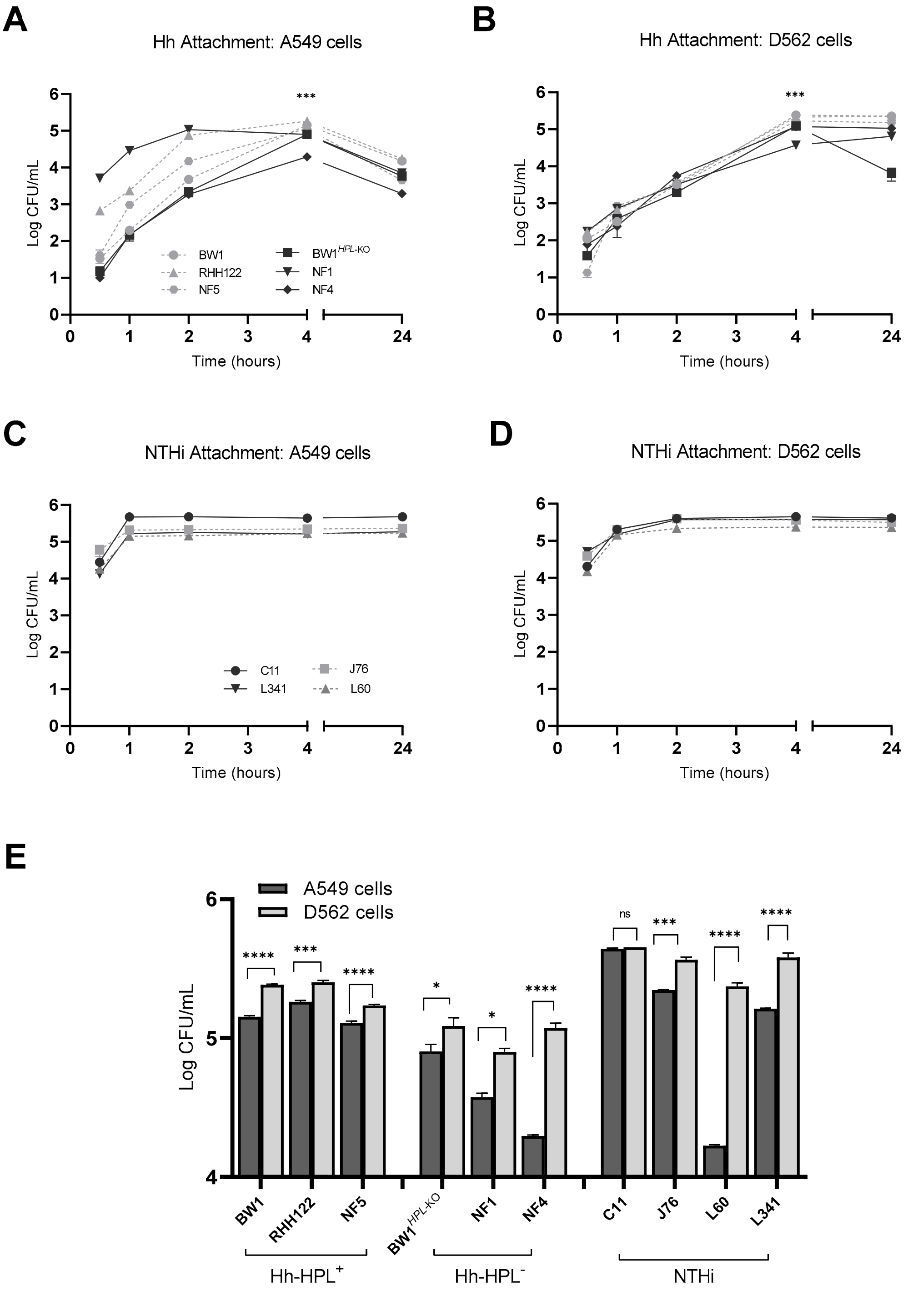

Figure S1| Baseline bacterial attachment to epithelial cells. Colony counts (CFU/mL) of Hh attached to A549 **(A)**, and D562 **(B)** cell monolayers, showing significant variation between Hh strains. Colony counts (CFU/mL) of NTHi to A549 **(C)**, and D562 **(D)** during a 24-hour incubation period. A summary of Hh and NTHi attachment to A549 and D562 cell monolayers at maximum attachment time points is shown **(E).** Error bars represent the ±SEM or three biological replicates. ^{ns} not significant, * $p < 0.05$, *** $p < 0.001$, **** $p < 0.0001$.

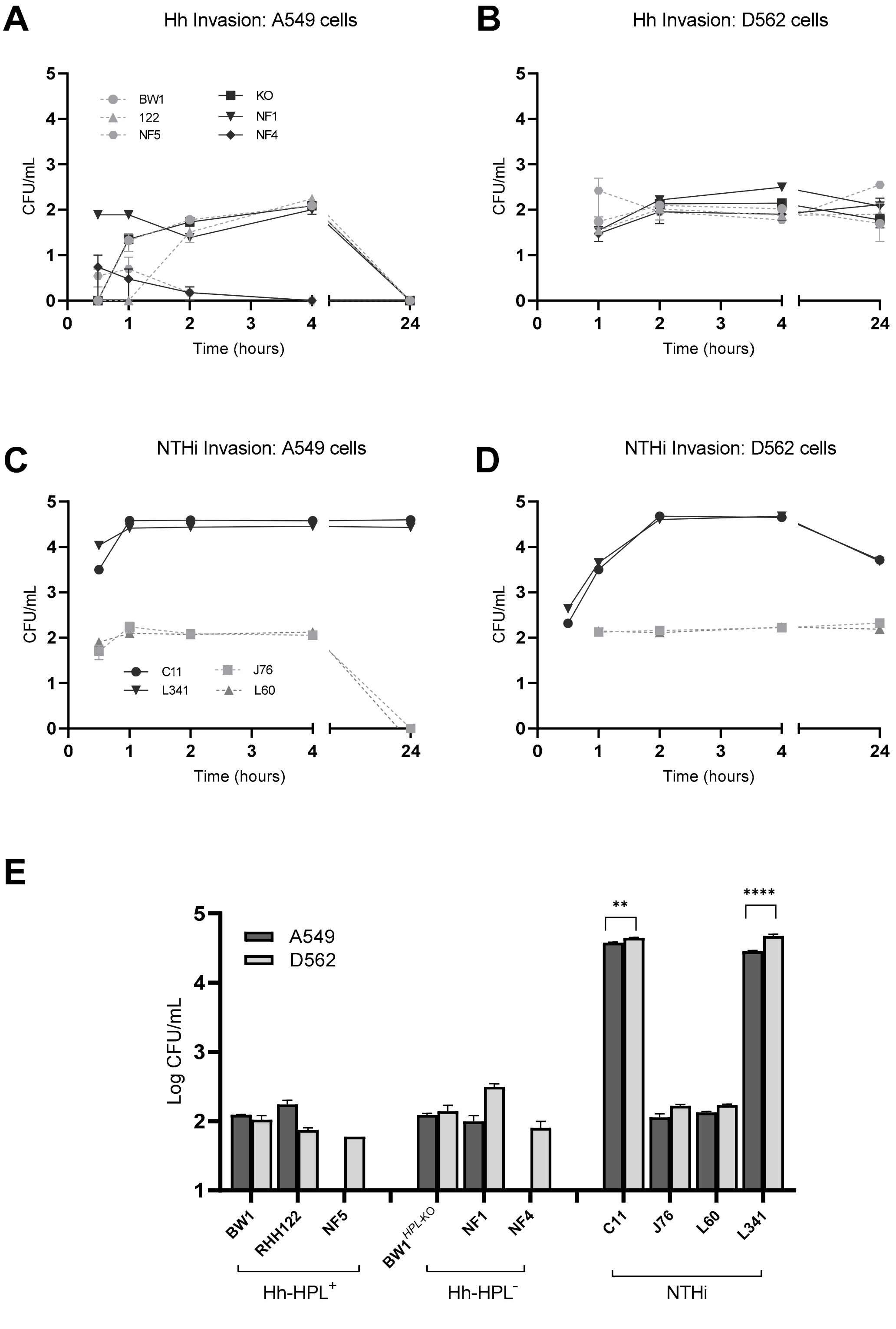

Figure S2| Baseline bacterial invasion of epithelial cells. Colony counts (CFU/mL) of internalised Hh in A549 **(A)**, and D562 **(B)** cell monolayers, and NTHi in A549 **(C)**, and D562 **(D)** during a 24-hour incubation period. A summary of Hh and NTHi invasion of A549 and D562 cell monolayers at maximum invasion time points is shown **(E).** Error bars represent the ±SEM or three biological replicates. ^{ns} not significant, * $p < 0.05$, ** $p < 0.05$ *** $p < 0.001$, **** $p < 0.0001$.

Table S1| Specific Hh treatment load (CFU/mL) for competitive colonisation experiments, standardised for differences in attachment capacity.

Hh strain	Treatment load for A549 cells (CFU/mL)	Treatment load for D562 cells (CFU/mL)
BW1	1.50×10^5	2.50×10^5
BW1$^{HPL\text{-}KO}$	3.00×10^5	5.00×10^5
RHH122	1.50×10^5	2.50×10^5
NF1	3.00×10^6	1.25×10^6
NF4	1.50×10^5	5.00×10^5
NF5	1.50×10^5	5.00×10^5

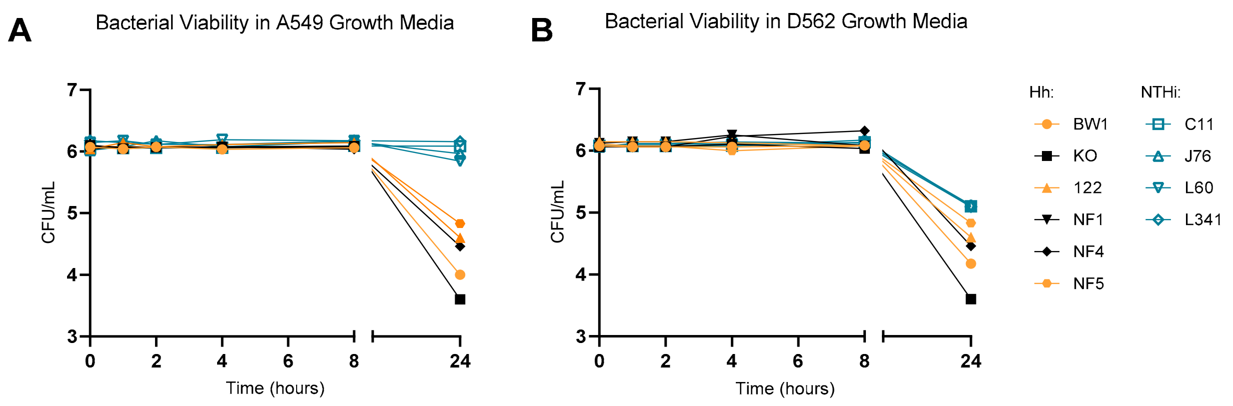

Figure S3| Bacterial viability in cell culture media. Colony counts (CFU/mL) Hh and NTHi strains grown in RPMI (A549 growth media) **(A)**, and EMEM (D562 growth media) **(B)** over 24 hours. Error bars represent the ±SEM of two biological replicates.

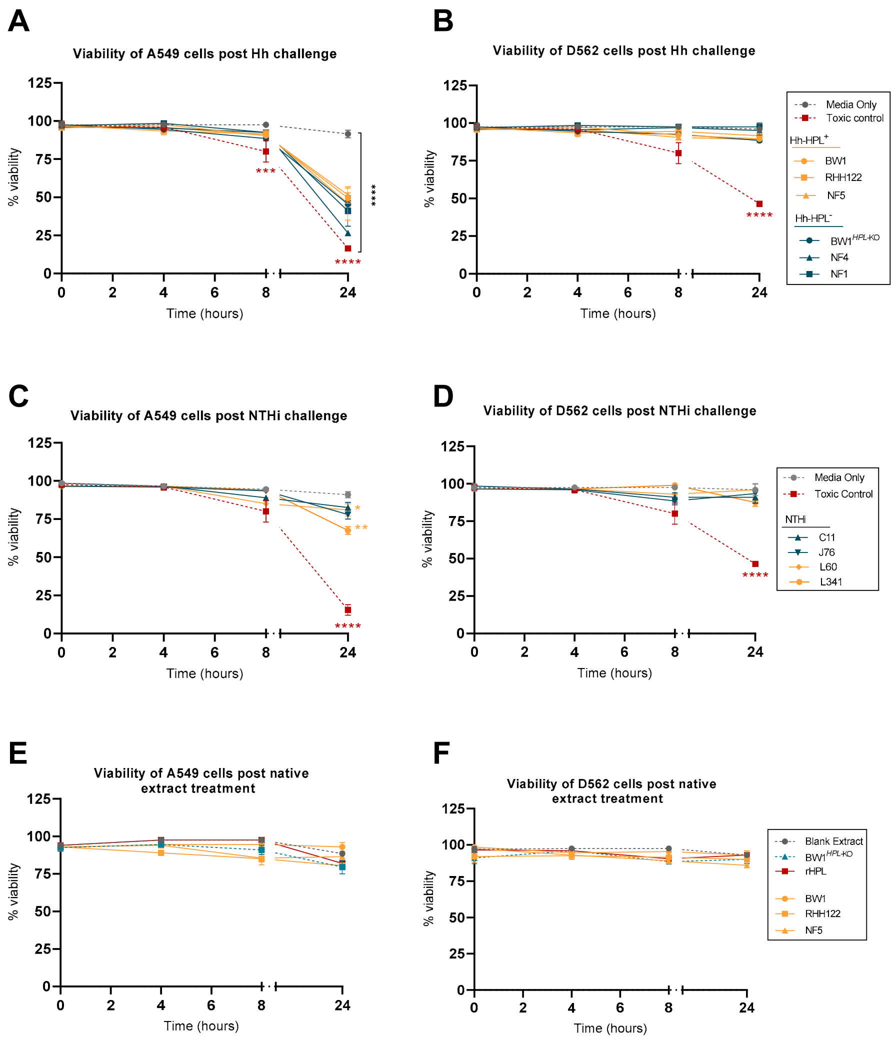

Figure S4| Viability of respiratory epithelium post bacterial challenge. Effect of 24-hour Hh challenge on A549 **(A)**, and D562 **(B)** cell viability, compared to media alone. Effect of 24-hour NTHi challenge on A549 **(C)**, and D562 **(D)** cell viability, compared to media alone. Effect of 24-hour HPL treatment on A549 **(E)**, and D562 **(F)** cell viability, compared to media alone. Error bars represent the ±SEM of three biological replicates. * $p < 0.05$, ** $p < 0.005$, **** $p < 0.0001$.

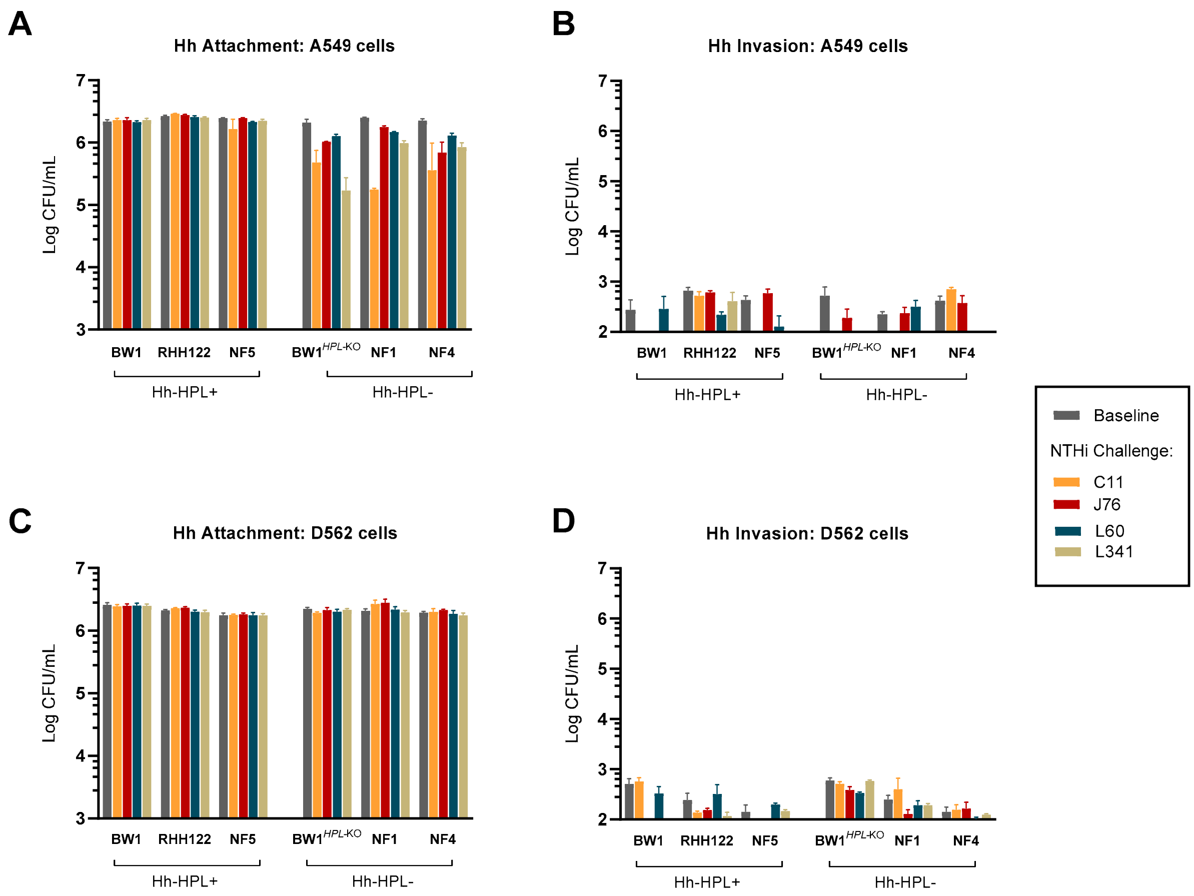

Figure S5| Hh attachment and invasion of A549 and D652 cells post NTHi challenge. Colony counts (CFU/mL) of Hh attached to A549 **(A)** and D562 **(B)** cell monolayers post NTHi challenge. Colony counts (CFU/mL) of internalised Hh after exposure to A549 **(C)** and D562 **(D)** cell monolayers post NTHi challenge. Error bars represent the ±SEM of three biological replicates, measured triplicate. *p<0.05, **** p < 0.0001.

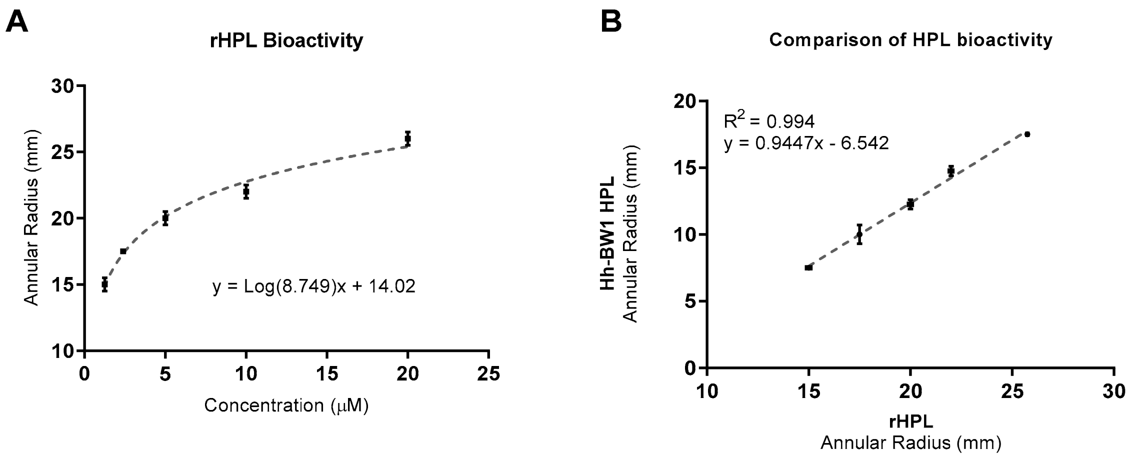

Figure S6|Bioactivity of recombinant and native HPL. Annular radius of inhibitory zones produced by serial 2-fold dilutions of rHPL on agar containing indicator strain NTHi-252 **(A)**. Linear regression comparing the relationship between concentration and anti-NTHi inhibitory activity of rHPL and nHPL from Hh-BW1 **(B)**.
